# Blend Structure and n-Type Thermoelectric Performance of PA6/SAN and PA6/PMMA Blends Filled with Singlewalled Carbon Nanotubes

**DOI:** 10.3390/nano11051146

**Published:** 2021-04-28

**Authors:** Beate Krause, Alice Liguoro, Petra Pötschke

**Affiliations:** Leibniz-Institut für Polymerforschung Dresden e.V. (IPF), Hohe Str. 6, 01069 Dresden, Germany; krause-beate@ipfdd.de (B.K.);

**Keywords:** polymer composites, blends, melt-mixing, carbon nanotubes, thermoelectrics, n-type behavior, rheology, morphology, segregated structures

## Abstract

The present study investigates how the formation of melt-mixed immiscible blends based on PA6/SAN and PA6/PMMA filled with single walled nanotubes (SWCNTs) affects the thermoelectric (TE) properties. In addition to the detailed investigation of the blend morphology with compositions between 100/0 wt.% and 50/50 wt.%, the thermoelectric properties are investigated on blends with different SWCNT concentrations (0.25–3.0 wt.%). Both PA6 and the blend composites with the used type of SWCNTs showed negative Seebeck coefficients. It was shown that the PA6 matrix polymer, in which the SWCNTs are localized, mainly influenced the thermoelectric properties of blends with high SWCNT contents. By varying the blend composition, an increase in the absolute Seebeck coefficient, power factor (PF), and figure of merit (ZT) was achieved compared to the PA6 composite which is mainly related to the selective localization and enrichment of SWCNTs in the PA6 matrix at constant SWCNT loading. The maximum PFs achieved were 0.22 µW/m·K^2^ for PA6/SAN/SWCNT 70/30/3 wt.% and 0.13 µW/m·K^2^ for PA6/PMMA/SWCNT 60/40/3 wt.% compared to 0.09 µW/m·K^2^ for PA6/3 wt.% SWCNT which represent increases to 244% and 144%, respectively. At higher PMMA or SAN concentration, the change from matrix-droplet to a co-continuous morphology started, which, despite higher SWCNT enrichment in the PA6 matrix, disturbed the electrical conductivity, resulting in reduced PFs with still increasing Seebeck coefficients. At SWCNT contents between 0.5 and 3 wt.% the increase in the absolute Seebeck coefficient was compensated by lower electrical conductivity resulting in lower PF and ZT as compared to the PA6 composites.

## 1. Introduction

The growing importance of thermoelectrics is reflected in the fact that this form of energy harvesting is finding its way into more and more areas [1,2,3]. In solving global energy problems, thermoelectric (TE) solutions are also playing an increasing role. In the last decade, organic thermoelectric materials and devices are gaining importance and are extensively studied. Among them, intrinsically electrically conductive polymers (ICPs) and their composites play the major role [4,5,6,7,8,9,10], however, also conductive polymer composites (CPCs) consisting of an insulating polymer matrix and electrically conductive fillers are increasingly studied [10]. Whereas most examples concern solution mixing as the processing method for such composites [10,11,12,13], in context with industrial applications, especially those based on thermoplastic polymers processed by melt-mixing methods are of interest [14,15,16,17]. Such CPC materials are, compared to the typically used materials like Bi_2_Te_3_, cost-effective, lightweight, nontoxic, without solvent use, easy to shape in different geometries and device constructions, and obtainable without geopolitical risks. However, still they show much lower thermoelectric values and efficiency compared to ICPs and traditional TE materials.

Carbon nanotubes (CNTs) are a suitable material group to obtain electrical conductivity in such CPCs at low loadings. This is due to the very high electrical conductivity and aspect ratio of this carbon material [18]. The thermoelectric properties of CNTs were comprehensively studied in literature [10,19,20,21,22]. Thus, CPCs with multiwalled CNTs (MWCNTs) or singlewalled CNTs (SWCNTs) are promising for TE materials. Such carbon nanostructures were for example shown to be very suitable to enhance the electrical performance of ICPs, such as PEDOT:PSS [23,24,25] and polyaniline (PANI) [26,27,28]. Next to CNTs, in CPCs also carbon nanofibers (CNFs) were studied [29,30]. Whereas most of the studied commercial CNT materials show p-type conduction behavior with positive Seebeck coefficients (S) [31,32,33] due to oxygen groups formed at their surface at defect sites [34,35], for commercial CNF materials n-type conduction behavior with slightly negative S-values was found [36]. Also, synthesis of CNTs containing nitrogen (N) results in negative S-values [37,38,39,40].

Interestingly, the incorporation of CNTs in different polymer matrices can result in conducting behavior that deviates from that of the starting CNTs. Whereas in melt-mixed composites based on polypropylene (PP), poly(butylene terephthalate) (PBT), poly(vinylidene fluoride) (PVDF) or polycarbonate (PC) all composites, irrespective of the selection of the CNT grade, showed positive S-values, incorporation of SWCNTs in different polyamide (PA) types and acrylonitrile butadiene styrene (ABS) led to negative S-values [33]. This implies that p- and n-type behavior, both of which are needed to construct thermoelectric modules, can be designed by proper selection of CNT and polymer type. When incorporating CNFs or N-doped CNTs into PP or PBT, the composites showed negative S-values as well [33,38].

Among the examples for negative Seebeck coefficients, the combination of polyamide 6 (PA6) with SWCNTs is especially interesting. For this polymer matrix it was found that despite the positive S-value of the nanotubes the incorporation of SWCNT Tuball^TM^ leads to negative Seebeck coefficient (e.g., −47 μV/K at 5 wt.% loading). In contrast, PA6 composites with HiPco SWCNT Unidym resulted in lower S-values than the SWCNT material but still with a positive sign [33]. This doping effect was explained by an electron donation of the amide group, which has one free electron pair at nitrogen and two free electron pairs at oxygen, to the SWCNTs. Such polymer induced doping to SWCNTs was also discussed by other authors [41].

It was the aim of this study to use PA6 in conductive SWCNT containing immiscible blends with other polymers and to study the influence of such blending on the n-type thermoelectric behavior. Polymer blends have broad applications in materials science [42] and recent examples show e.g., the use in membranes in demanding environments [43], in self-healing applications [44], for highly antibacterial materials [45], for drug-delivery [46] and for vapor-sensing applications [47]. By combining polymer blends with CNTs, the distribution and density of the nanotube network can be modified, especially if the nanotubes localize preferentially in one of the components. This is in most immiscible blends the case, as the filler typically has a thermodynamic tendency to localize in the better wetting component [48,49,50,51,52]. This localization can be interacted by sterical hindrance in case of platelet like fillers or agglomerates [50,53] or by kinetic reasons, like too short mixing time or too high viscosity of the components hindering the filler migration to this better wetting component [54,55,56]. Even if nanotubes are first localized in the worse wetting component of a blend and then mixed with the thermodynamic preferred component, their migration occurs very fast, as shown on MWCNTs in PC/poly(styrene-*co*-acrylonitrile) (SAN) blends. Premixed MWCNTs in SAN migrated toward PC within the first 60 s of mixing [56].

For the purpose to make blends based on PA6, two amorphous polymer partners, namely SAN and poly(methyl methacrylate) (PMMA) were selected differing in their melt viscosity. Both polymers were expected to be the thermodynamically less preferred component for SWCNT localization in such blends. Thus, the assumed SWCNT localization in PA6 enhances the local content in this component by creating at the same time a different pattern of the conductive areas depending on the blend morphology. This can be regarded as a segregated SWCNT structure, a concept shown to be effective for the thermoelectric properties of poly(vinyl acetate) (PVAc) with SWCNTs [57] and ultrahigh molecular polyethylene (UHMPE) combined with MWCNTs and Bi_2_Te_3_ [58]. Whereas in the references the segregated CNT network was formed at the interphase between neighboring PVAc or UHMPE particles, in our case the segregation is within the PA6 matrix of the blend structures. By changing the blend ratio, different morphologies were aimed. Two sets were compared: either 3 wt.% SWCNT was the summary content in the blend, or the SWCNT content was chosen such a way that 3 wt.% SWCNT could always be assumed in the PA6 component. For comparison, pure PA6 filled with 3 wt.% SWCNT and 6 wt.% was also studied. Next to the Seebeck coefficient S and electrical conductivity σ also the power factor ($PF=S^{2}\sigma$) and the figure of merit ($ZT=S^{2}\sigma T\kappa^{-1},$ with κ as thermal conductivity and T as temperature) were calculated. The blend composites were also characterized concerning their state of SWCNT dispersion, blend morphology type and fineness, and thermal conductivity. The localization behavior of the SWCNTs was studied and compared with the theoretical expectations.

## 2. Materials and Methods

### 2.1. Materials

**Figure 1:**
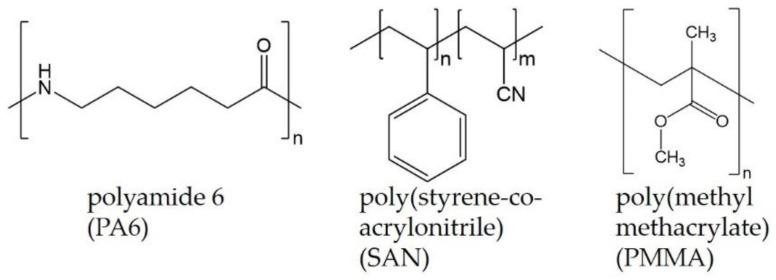
Chemical structure of the polymers PA6, SAN, and PMMA.

As polymers polyamide 6 (PA6) Ultramid^®^ B27E (BASF SE, Ludwigshafen, Germany), poly(styrene-*co*-acrylonitrile) (SAN) Luran 358N (INEOS Styrolution Group GmbH, Frankfurt am Main, Germany), and poly(methyl methacrylate) (PMMA) Plexiglas 8N (Evonik Industries AG, Essen, Germany) were used. The densities of pure polymers are 1.12 g/cm^3^ (PA6), 1.05 g/cm^3^ (SAN), and 1.11 g/cm^3^ (PMMA) and the densities of the blends are given in context with the thermal conductivity measurement in chapter 3.5. The chemical structure of the three polymers is illustrated in Figure 1.

As filler singlewalled carbon nanotube (SWCNT) material Tuball^TM^ (OCSiAl S.a.r.l., Luxembourg, Luxembourg) with a nanotube content of 75% was used having a mean diameter of 1.6 nm and lengths of more than 5 µm [59].

### 2.2. Composite Preparation

Blends of PA6 and SAN or PMMA in ratios 90 wt.%:10 wt.% up to 50 wt.%:50 wt.% and their composites were prepared by melt compounding in a one-step mixing procedure. PMMA and SAN are both amorphous polymers with Vicat softening temperatures of 108 °C and 106 °C, respectively. As PA6 only melts at around 220 °C, the SWCNTs are therefore first wetted with PMMA or SAN. During the melt mixing time, the SWCNTs can localize in the preferred component. Two sets of blends were produced whereby all blend ratios are related to weight %. In set 1 (Table 1), all blends contain 3 wt.% SWCNT. In the second set (Table 1), the SWCNT content was selected so that there is always 3 wt.% SWCNT in the PA6 component, assuming complete SWCNT localization in PA6. For comparison, also pure PA6 was filled with 3 and 6 wt.% SWCNT. In addition, in 50/50 blends, lower SWCNT amounts were used.

Melt compounding was performed in a conical twin-screw microcompounder DSM15 (Xplore Instruments BV, Sittard, The Netherlands) having a volume of 15 ccm, at 280 °C for 5 min and a rotation speed of 250 rpm. The materials were pre-mixed in the dry state and fed to the compounder. The extruded strands were cut into pieces and compressing molded to plates (diameter of 30 mm and thickness of 0.3 mm) using a hot press PW40EH (Paul-Otto-Weber GmbH, Remshalden, Germany) at 280 °C with a pressing time of 1.5 min. Strips (40 mm × 5 mm × 0.3 mm) were cut from the plates to measure electrical resistance and thermoelectric properties. Plates with a diameter of 12.3 mm and a thickness of 2 mm were prepared for measurements of the thermal conductivity. For rheological measurements plates with a diameter of 25 mm and 2 mm thickness were compression molded.

### 2.3. Material Characterizations

For the investigation of CNT macrodispersion in the blends by transmission light microscopy (LM), thin cuts (thickness 5 µm) were prepared from the extruded strands with a microtome RM2265 (Leica MikrosystemeVertrieb GmbH, Bensheim, Germany) equipped with a diamond knife at room temperature. The cuts were fixed on glass slides using the aqueous mounting medium Aquatex^®^ (Sigma-Aldrich, Steinheim, Germany). The LM investigations were performed with a microscope BX53M combined with a camera DP74 (Olympus Deutschland GmbH, Hamburg, Germany). The agglomerate area ratio A_A_ (%), defined as the ratio between the area of filler agglomerates and the total area of the imaged sample, was calculated to quantify the remaining MWCNT agglomerates in the blends. The mean values and standard deviations of agglomerate area ratio were calculated from at least ten images taken at the magnification indicated in the LM images.

Scanning electron microscopy (SEM) images of the samples were acquired using an ULTRA Plus (Carl Zeiss AG, Oberkochen, Germany) scanning electron microscope at 3 kV acceleration voltage and applying the SE2 detector. Cut surfaces of strands and compressing molded plates were used with the cutting conditions named above. To facilitate the observation of the blend structure, the second component was dissolved. Chloroform was used to dissolve the SAN and acetone dissolved the PMMA component. The samples were immersed in the solvents for one night, followed by two hours of drying at 40 °C to evaporate the solvent. All samples were sputtered with a 3 nm platinum film. For the calculation of the mean diameters of the pores (dissolved PMMA and SAN) an image area of 35,148 µm^2^ was observed (four images at the magnification shown) using the software Stream Motion 2.3.3 (Olympus Deutschland GmbH, Hamburg, Germany). Thereby, the area equivalent circle diameter was used; the standard deviations relate to the four images taken per sample. For blends with the weight ratio of 50/50, such quantification was only possible for blends without SWCNTs, as in the case of the compression-molded plate of the PA6/SAN = 60/40 wt.% blend, since these blends tend to have co-continuous structures.

The thermal conductivity of the composites was calculated from the product of thermal diffusivity, density, and specific heat capacity. The thermal diffusivity was measured on round samples (diameter 12.3 mm, thickness 2 mm) through the plate thickness using the light flash apparatus LFA 447 NanoFlash (Netzsch-Gerätebau GmbH, Selb, Germany) at 25 °C. The specific heat capacity of the composites was calculated by comparing the signal heights between the composite and the reference Pyroceram 9606 (with known specific heat capacity) using the LFA 447 NanoFlash. The density of composites was determined using a buoyancy method.

The thermoelectric characterization was carried out in a Seebeck measuring device developed at IPF Dresden. More details are given in [33]. The measuring temperature was set to 313.2 K (40.0 °C), with eight temperature variations up to ±8 K. The mean values and standard deviations of the Seebeck coefficient were calculated from the 16 values resulting from the measurement on two specimens with eight thermovoltage measurements each. The measurements of the electrical volume resistivity were done using the same equipment applying the 4-wire technique on the same two samples. The free sample length between the silver coated ends was 12 mm (width around 5 mm, thickness around 0.3 mm).

**Figure A1:**
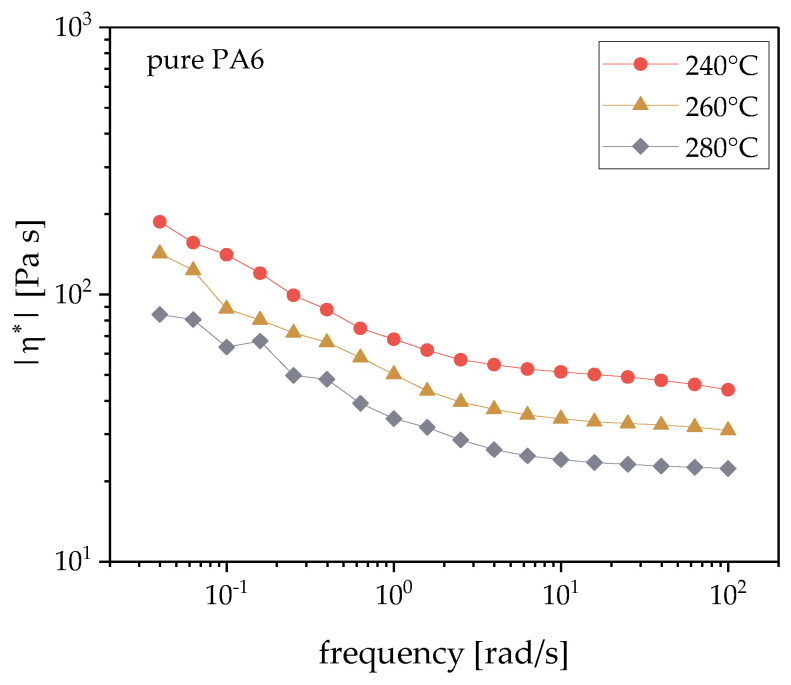
Complex viscosity |η*| of the pure polyamide 6 at 240 °C, 260 °C and 280 °C.

Melt rheological properties in the oscillation mode were obtained for the blend components and selected blends. An ARES oscillation rheometer (TA Instruments, New Castle, DE, USA) was used and the measurements were carried out under nitrogen atmosphere at 240 °C with a parallel plate geometry (diameter 25 mm, gap approximately 1 mm). Dynamic frequency sweeps (strain 5%) with increasing and decreasing frequency (between 0.4 and 100 rad/s) were used, whereby the second sweep was used for interpretation. The values of the complex melt viscosity |η*| are discussed. The measuring temperature of 240 °C was set lower than the processing temperature of 280 °C in order to reduce the influence of temperature on the blend structure, i.e., prevent blend coarsening during the rheological measurements. For comparison, also the polymers were measured at 240 °C. In addition, pure polyamide 6 was tested at 260 °C and 280 °C as shown in Appendix Figure A1.

## 3. Results

### 3.1. Rheological Characterization of the Blend Components and Blends

**Figure 2:**
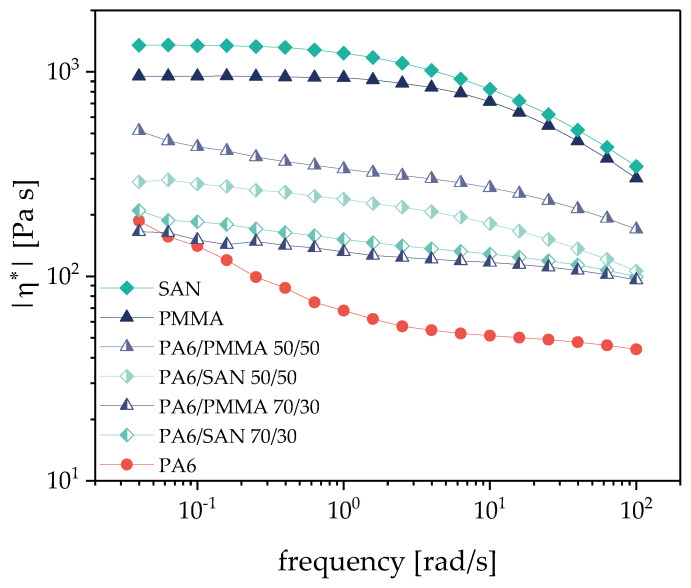
Complex viscosity |η*| of the blend components and selected unfilled blends at 240 °C.

The blend morphology of an immiscible blend is dependent on the melt rheological parameters of its constituents. Therefore, the complex viscosity |η*| was determined (Figure 2). Even if the measurement temperature is lower than the used processing temperature, it can be clearly seen that PA6 shows a much lower melt viscosity over the whole frequency range and that SAN has a higher viscosity than PMMA. Based on the values at 100 rad/s, viscosity ratios of PA6/SAN of 0.13 and of PA6/PMMA of 0.15 can be calculated.

Interestingly, the 50/50 wt.% blends show higher values for the blend with PMMA compared with SAN, whereas in blends with 30 wt.% of the second component the blends with SAN shows higher values than that with PMMA. The viscosity values are between those of the components (Figure 2). Based on the general tendency that the phase inversion concentration in blends depends on the viscosity ratio and the lower viscosity components tends to form the matrix in immiscible blends, these viscosity relationships suggest that PA6 has the tendency to form the continuous component even at lower contents than 50 vol.% (53.3 wt.% with SAN, 50.5 wt.% with PMMA) if no SWCNTs are added.

**Figure 3:**
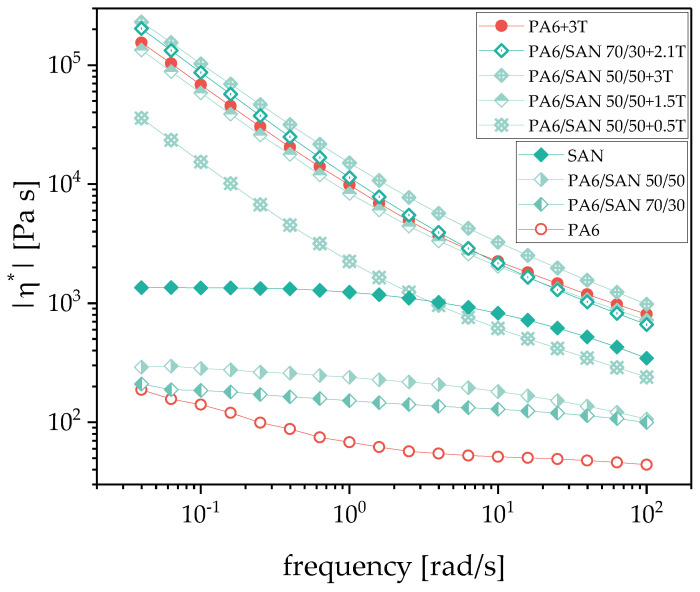
Complex melt viscosity |η*| of PA6, SAN, and PA6/SAN blends without and with different amounts of SWCNT filler Tuball (T).

However, the blends under investigation here contain SWCNTs that are expected to reinforce the PA6 component of the blend. Comparing the viscosity curves of pure PA6 and that of PA6 filled with 3 wt.% SWCNTs (Figure 3), a dramatic increase in melt viscosity is seen especially at low measurement frequencies, and the filled PA6 exceeds the viscosity values of pure SAN and PMMA. Thus, in the filled blends the viscosity ratios between PA6 with 3 wt.% filler and the second polymer get values larger than one. With viscosity ratios at 100 rad/s of 2.34 for SAN and 2.68 for PMMA as blend partners it is expected that SAN and PMMA tend to form the matrix even at higher PA6 contents than 50 vol.%. Based on the Tailor equation [60,61] it may be also expected that due to the higher viscosity ratio, the matrix-droplet morphologies of the PA6/PMMA blends will have smaller particle sizes than the blends with SAN assuming comparable interfacial tension and shear conditions.

**Figure 4:**
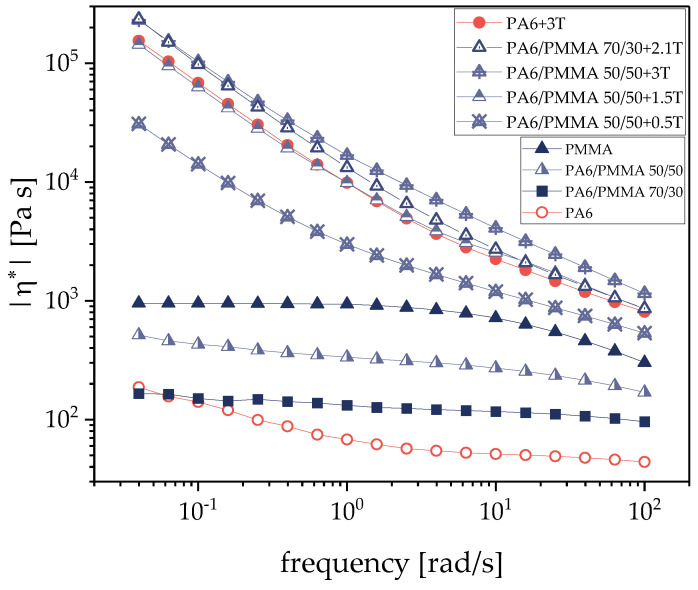
Complex melt viscosity |η*| of PA6, PMMA and PA6/PMMA blends without and with different amounts of SWCNT filler Tuball (T).

Figure 3 and Figure 4 show the frequency dependence of the complex viscosity for selected SWCNT filled PA6/SAN and PA6/PMMA blends, respectively, in comparison to their unfilled blends and pure and 3 wt.% filled PA6. Corresponding to the much-increased viscosity after filling pure PA6 with SWCNTs, also all blends with SWCNTs have much higher viscosity values than the unfilled blends and show a strong increase in complex melt viscosity when lowering the measuring frequency, similar to the behavior of filled PA6. Such strong shear thinning behavior is typical for composites above the rheological percolation of the filler [62,63]. For PA6/SAN = 50/50 wt.% blends the largest step is seen after addition of 0.5 wt.% to the whole blend, whereas the increase in SWCNT content to 1.5 and 3 wt.% shows fewer intensive increases resulting in values very similar but only slightly lower or even higher than the filled PA6. The exceeding of melt viscosity of PA6/SAN with 3 wt.% over the values of PA6 with 3 wt.% illustrates the additional effect of the interfaces within the blend morphology and implies a co-continuous structure. The behavior of the PA6/PMMA blends is similar to that of PA6/SAN. Despite a lower melt viscosity of PMMA compared to SAN, the blends with PMMA show higher viscosity values at the same SWCNT loading. The viscosity curves of PA6/PMMA = 50/50 wt.% blends with SWCNTs equal or exceed the curve of PA6 filled with 3 wt.% starting at 1.5 wt.% SWCNT addition, which corresponds to 3 wt.% SWCNT in the PA6 component. Similarly to PA6/SAN blends, this implies the formation of a co-continuous morphology type.

### 3.2. SWCNT Macrodispersion in Blends Characterized by Light Microscopy

**Figure 5:**
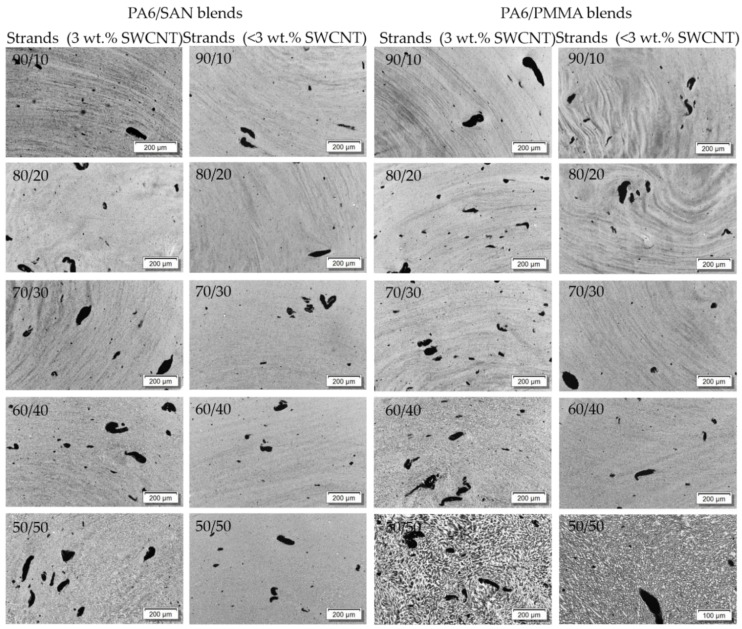
LM images of PA6/SAN and PA6/PMMA blends with different SWCNT contents for set 1 (3 wt.% SWCNT), for set 2 the SWCNT contents are given in Table 1.

For the purpose of characterization of SWCNT dispersion in the polymeric matrices, transmission light microscopy (LM) on thin sections is an adequate method. Selected blends of both blend systems were qualitatively (Figure 5) and quantitatively (Table 2) studied. It was found that in all blends a significant number of large agglomerates are visible. The stretched shape of the SWCNT bundles is typical for this kind of SWCNT and was shown before in PC [64], PP [64], PA6 [65], and ABS [65] based composites. The agglomerate area ratio as a measure of the SWCNT dispersion shows values of around 2.5% for the blends filled with 3 wt.% SWCNT. A dependency of the agglomerate area ratio on the blend ratio cannot be found. For the blends with lower SWCNT content, correspondingly lower values of the agglomerate area ratio were found.

**Figure 6:**
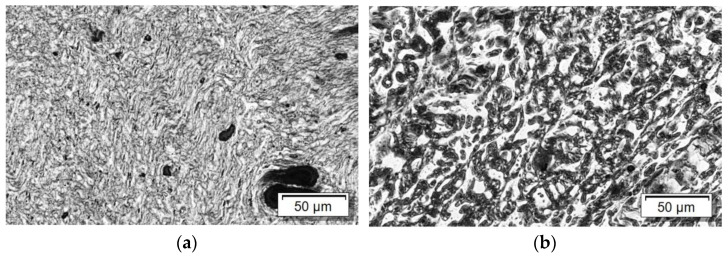
LM images of (**a**) PA6/SAN 50/50 + 3 wt.% SWCNT and (**b**) PA6/PMMA 50/50 + 3 wt.% SWCNT, at higher magnification.

Furthermore, the blend morphology can be identified from the LM images. In particular for the PA6/PMMA 50/50 composition, clearly lighter and darker areas can be recognized (Figure 5 and Figure 6b). The gray appearance can be related to the number of agglomerates with sizes equal to or slightly larger than the wavelength of visible light (ca. 400–700 nm) but smaller than visually detectable agglomerates [66,67]. The more nanotubes are dispersed in this size range, the darker an area in the image appears. With it the darker areas can be assigned to the component containing nanoscale-dispersed nanotubes. If areas appear brighter, fewer CNTs are dispersed in them. It can be concluded that the nanotubes are localized mainly in one component of the blend. Due to the 50/50 ratio of the PA6/PMMA blend, it is unfortunately not possible to determine in which component of the blend the CNTs are located. For PA6/PMMA 60/40 and PA6/SAN 50/50 (Figure 6b), irregularities are visible in the grey colors, but since the blend morphology is apparently very fine, it can only be deduced here that a selective CNT localization is present. The direct comparison of LM images at high magnification of PA6/PMMA 50/50 and PA6/SAN 50/50 (each with 3 wt.% SWCNT) in Figure 6 shows the differences in the blend morphology of the extruded strands which appears to be finer in case of SAN. It can therefore be concluded from the LM investigations that the type of the second polymer (PMMA or SAN) in the PA6 blends has an influence on the blend morphology.

As an example of characterization of the blend morphology by light microscopy, Göldel et al. [68] reported LM images of PC/SAN 60/40 blend that clearly show the selective localization of MWCNTs in the PC component. For the same blend system, Liebscher et al. [69] showed LM images of blends based on three types of polycarbonate with different viscosities. These images can be used to clearly distinguish the blend morphologies.

### 3.3. Blend Morphology Characterized by Scanning Electron Microscopy (Constant SWCNT Content of 3 wt.%)

**Figure 7:**
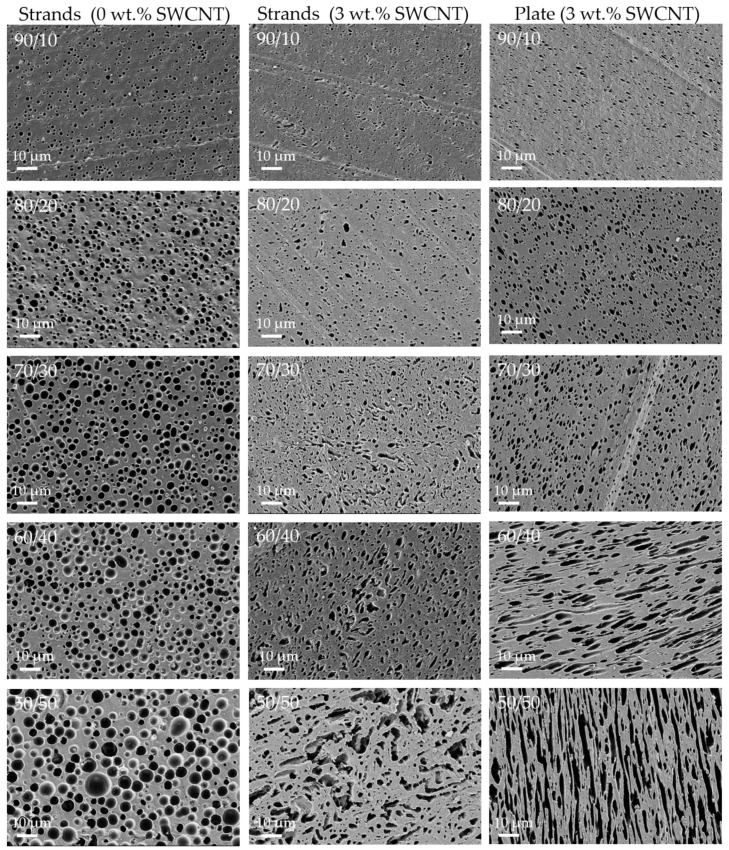
SEM images of etched flat surface of PA6/SAN blends with different blend ratio from 90/10 (**first line**) up to 50/50 (**last line**) and different kinds of sample such as strands without CNTs (**left**), strands with 3 wt.% CNTs (**middle**), and compression-molded plates with 3 wt.% CNTs (**right**).

**Figure 8:**
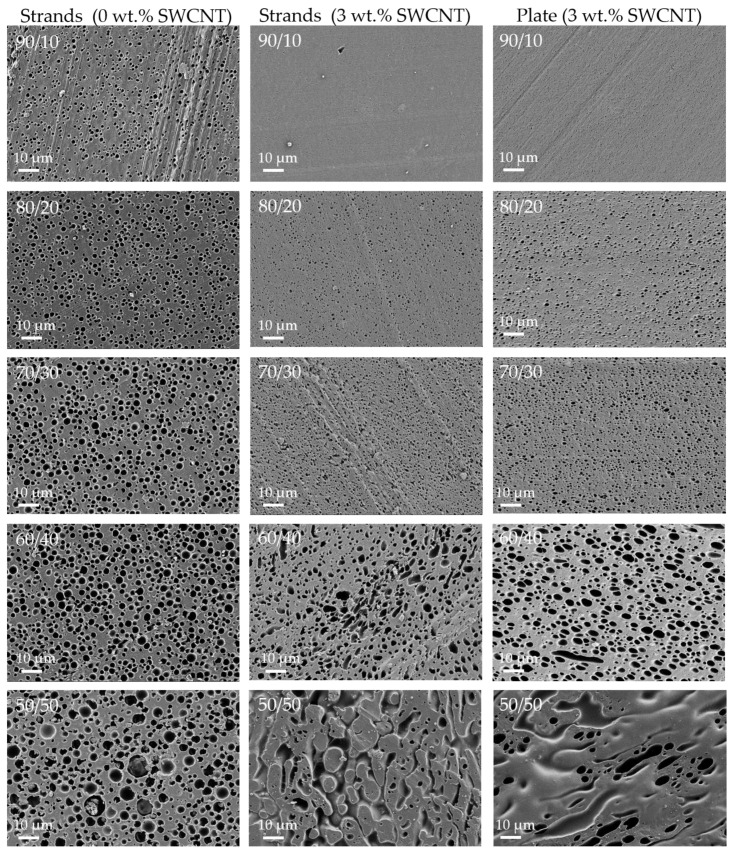
SEM images of etched flat surfaces of PA6/PMMA blends with different blend ratio from 90/10 (**first line**) up to 50/50 (**last line**) and different kinds of sample such as strands without CNTs (**left**), strands with 3 wt.% CNTs (**middle**), and compression-molded plates with 3 wt.% CNTs (**right**).

**Figure A2:**
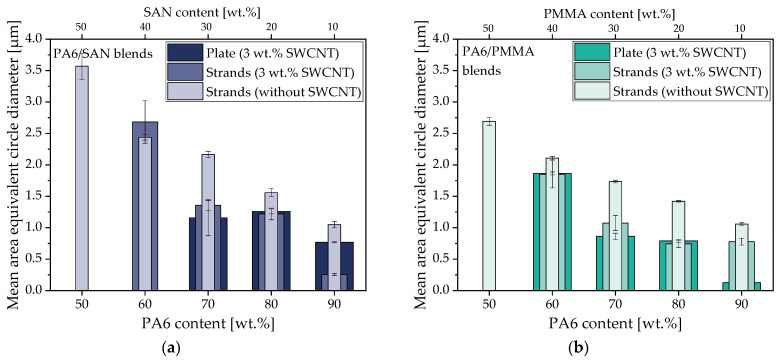
Mean area equivalent circle diameter of the dispersed (dissolved) components determined from SEM images shown in Figure 7 for PA6/SAN blends (**a**) and in Figure 8 and PA6/PMMA blends (**b**) such as strands without SWCNTs, strands with 3 wt.% SWCNTs, and compression-molded plates with 3 wt.% SWCNTs.

To characterize the blend morphology by SEM, the minor component (PMMA or SAN) was dissolved from the blend. This procedure was applied on flat surfaces of strands (with and without CNTs) as well as compression-molded plates. The SEM images are summarized in Figure 7 and Figure 8. In order to characterize the size of the dispersed particles of the minor component, the mean area equivalent circle diameters of the pores were determined and are shown in Figure A2.

A general trend is that the size of the distributed minor component increases with increasing proportion. This applies to extruded strands (1 µm up to 2.7 µm (PMMA) or 1–3.6 µm (SAN)) and extruded strands with CNTs (0.8–1.8 µm (PMMA), 0.3–2.7 µm (SAN) as well as to compression-molded plates with CNTs (0.1–1.9 µm (PMMA), 0.8–1.2 µm (SAN)). For the same blend ratio, CNT content and sample preparation, the disperse component is always smaller when PMMA is used instead of SAN. This is in accordance with the expectations based on the slightly lower melt viscosity of PMMA compared to SAN.

On the one side, the comparison of strands with CNTs and without CNTs shows the influence of CNT incorporation on the blend morphology. It was found that the size of the dispersed component decreases with CNT incorporation for the blend ratios 90/10 wt.% up to 60/40 wt.%. For the 50/50 wt.% blends a significant change of the blend structure is visible. In blends without CNTs, the SAN- or PMMA-dissolved component appears as spherical areas. With the addition of CNTs, these areas change to a more elongated shape, which suggests a co-continuous blend morphology. For comparison, Bose et al. [70] described for PA6/ABS 50/50 wt.% blends a refinement of the remaining PA6 component in the blends with increasing CNT content (ABS was removed by etching). Also for PC/SAN 60/40 wt.% [69], PC/ABS 45/55 wt.% [71], or PC/poly(vinylidene fluoride) (PVDF) 40/60 wt.% [47] blends it was described that after the incorporation of MWCNTs the blend morphology became even finer resulting in an increase of the area of blend interface.

On the other side, the comparison of strands and compression-molded plates each with 3 wt.% SWCNT shows the influence of the compression-molding step on the blend morphology. This influence has to be considered, since the characterization of the thermoelectric properties as well as the thermal conductivity measurements was always carried out on compression-molded samples. After compression molding the blend structure is only slightly changed. The shape and size of the dispersed component is nearly the same for PA6/SAN and PA6/PMMA with ratios between 90/10 wt.% and 70/30 wt.%. For blends with the blend ratio of 60/40 wt.% the shape of dispersed component switched from spherical in the strands to elongated in the plates, which is more pronounced in case of PA6/SAN blends. For the 50/50 wt.% blends in both blends and sample shapes the co-continuous structures are visible, however the appearance is different.

### 3.4. CNT Localization in the Blends

In the literature, the localization of fillers in polymer blends is usually explained by the general tendency of interfacial energy minimization, which leads to a more or less pronounced driving force to arrange the fillers in the energetically preferred blend component.

In the case of the three component system PA6/SAN/CNT and PA6/PMMA/CNT, the interfacial tensions were calculated from surface tensions and their disperse and polar parts, using both the harmonic-mean and the geometric-mean equation [72]. The harmonic-mean equation is commonly used to calculate the interfacial tension *γ*_12_ between two components:

(1) $$\gamma_{12}=\gamma_{1}+\gamma_{2}-4\left[\frac{\gamma_{1}^{d}\gamma_{2}^{d}}{\gamma_{1}^{d}+\gamma_{2}^{d}}+\frac{\gamma_{1}^{p}\gamma_{2}^{p}}{\gamma_{1}^{p}+\gamma_{2}^{p}}\right]$$

with *γ*_1_ and *γ*_2_ the surface energy of the components 1 and 2, *γ*^d^_1_ and *γ*^d^_2_ the disperse part of the surface energy of components 1 and 2, and *γ*^p^_1_ and *γ*^p^_2_ the polar part of the surface energy of components 1 and 2.

The geometric-mean equation is described in the literature as being more suitable in comparison to the harmonic-mean equation for high surface tension [72]:

(2) $$\gamma_{12}=\gamma_{1}+\gamma_{2}-2\left(\sqrt{\gamma_{1}^{d}\gamma_{2}^{d}}+\sqrt{\gamma_{1}^{p}\gamma_{2}^{p}}\right)$$

with *γ*_1_ and *γ*_2_ the surface energy of the components 1 and 2, *γ*^d^_1_ and *γ*^d^_2_ the disperse part of the surface energy of components 1 and 2, and *γ*^p^_1_ and *γ*^p^_2_ the polar part of the surface energy of components 1 and 2.

In Table 3 the surface tension values of all components of the two blend composite systems as calculated for 280 °C based on values taken from the literature (seeTable A1) are summarized. These surface tension values were applied for the calculation of interfacial tensions. In this study, SWCNTs were incorporated; however, there are no surface tension values for SWCNTs available in the literature. Thus, for a rough estimate, the values published for MWCNTs were used [73,74].

Based on the interfacial tension values between two components (Table 4), the wetting coefficient *ω*_a_ of the filled blend system can be calculated by an equation, which was derived from the Young’s Equation [79]:

(3) $$\omega_{a}=\frac{\gamma_{CNT-SAN}-\gamma_{CNT-PA6}}{\gamma_{PA6-SAN}};\;\omega_{a}=\frac{\gamma_{CNT-PMMA}-\gamma_{CNT-PA6}}{\gamma_{PA6-PMMA}}$$

where the indices of the interfacial tension values *γ* indicate the components of the system.

If the wetting coefficient is greater than 1, then the CNTs should be localized in the PA6 component. If the wetting coefficient *ω*_a_ is between −1 and 1, then the CNTs are expected to accumulate at the interphase. For values smaller than −1, localization of the CNTs in the SAN or PMMA component is predicted.

The calculated wetting coefficients (Table 5) for all systems using values of MWCNT1 [73] are clearly above 1. This means that the CNTs prefer the PA6 component as a matrix over SAN or PMMA. If values for MWCNT 2 [74] are included in the calculation, the wetting coefficient is approximately between 0 and 0.5. By following the interpretation according to Sumita et al. [79], this means that the CNTs localize at the interface. For the interpretation of the wetting coefficient, it has to be considered that only approximate values for CNTs were used in the calculation. However, based on the calculated values, it can be concluded that the CNTs tend to prefer the PA6 component or the interface as a place of presence. CNT localization in the SAN or PMMA component is therefore very unlikely.

**Figure 9:**
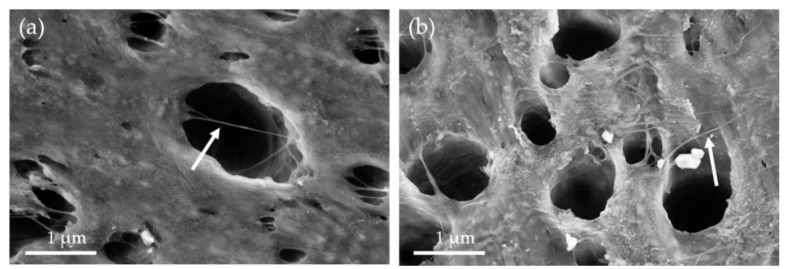
SEM images of flat surfaces of (**a**) PA6/SAN 90/10 + 3 wt.%SWCNT and (**b**) PA6/PMMA 80/20 + 3 wt.% SWCNT, the arrows indicate CNTs (blends were etched to remove SAN or PMMA).

These results correlate also with the SEM study on the blends. In Figure 9 high magnification images of blends filled with CNTs as selected examples are seen. The SAN or PMMA component was removed by etching. Polymer-covered CNTs are visible as light grey lines on the inner walls of the cavities. In addition, CNTs are located across the holes created by dissolving out SAN or PMMA. Both findings show that the CNTs were not removed together with the dissolved SAN or PMMA during etching. This underlines that the CNTs are localized in both blend types in the PA6 component.

### 3.5. Thermal Conductivity of Composite and Blend Material

In Table 6 the results of measurements of thermal conductivity on selected samples are summarized. The thermal conductivity of pure PA6 (0.31 W/m·K) was slightly higher compared to unfilled SAN (0.20 W/m·K) and PMMA (0.25 W/m·K). For the blends with 50/50 ratio a thermal conductivity of 0.27 W/m·K was determined which is between the values of the single polymers. As expected, the addition of 3 wt.% SWCNT to the polymers leads to an increase in thermal conductivity, with only 0.45–0.48 W/m·K achieved for the 50/50 blends compared to 0.62 W/m·K for the PA6/SWCNT composite.

### 3.6. Thermoelectric Measurements on the Blend Composites

#### 3.6.1. Blends with 3 wt.% SWCNT Content

As reported by Kunz et al. [65] the electrical percolation threshold of the SWCNT Tuball™ in PA6 is below 0.1 wt.% SWCNT. Thus, the selected SWCNT concentration in the blends is significantly above the electrical percolation threshold. It can be concluded that a very dense and stable conductive nanotube network is formed in the blends, especially under the consideration that the CNTs are localized in the PA6 component. Thus, depending on the ratio of the blend components, an even higher local CNT concentration is reached in the PA6 component of the blends, which goes up to 6 wt.% in the 50/50 blends.

**Figure 10:**
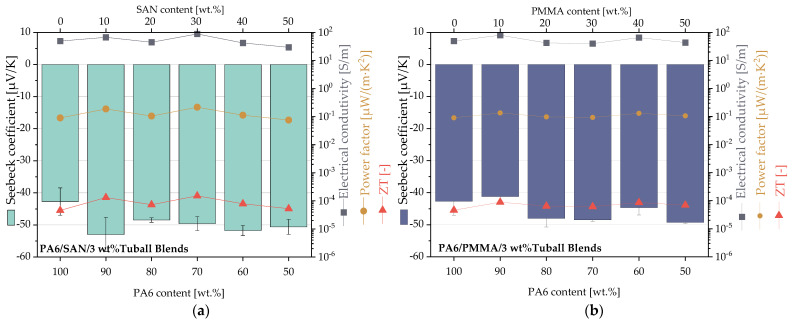
Thermoelectric parameters of (**a**) PA6/SAN/3 wt.% SWCNT blends and (**b**) PA6/PMMA/3 wt.% SWCNT blends having different ratios of the blend components (values are given in Table A2 and Table A3).

The thermoelectric properties of PA6/SAN and PA6/PMMA blends with different polymer blend ratios at a constant SWCNT content of 3 wt.% in the blends are shown in Figure 10. The corresponding values of the thermoelectric parameters of PA6/SAN/3 wt.% SWCNT blends and PA6/PMMA/3 wt.% SWCNT blends are listed in Table A2 and Table A3.

The TE results in Figure 10 show negative Seebeck coefficients as well for the PA6 composite as both blend systems with 3 wt.% SWCNT. The PA6/3 wt.% SWCNT composite has a value of −42.7 μV/K. This value tends slightly more negative if the content of the second blend component increases. Thereby the type of the second component, SAN or PMMA, does not play a significant role. The highest negative Seebeck coefficient was found with −53.0 μV/K for PA6/SAN/SWCNT = 90/10/3 wt.%. For both blend systems, the electrical conductivity values fluctuate slightly and reach values between 29.5 and 88.9 S/m, without a clear trend. For power factor and ZT, the calculated values are almost around 0.1 µW/m·K^2^ and 2 × 10^−4^, respectively.

#### 3.6.2. Blends with Constant SWCNT Content of 3 wt.% in the PA6 Component

Since the SWCNTs are localized in the PA6 component, in the second set the CNT content was chosen in such a way that there are always 3 wt.% SWCNT in the PA6 component of the blend. This should ensure that comparable network densities are achieved in PA6.

**Figure 11:**
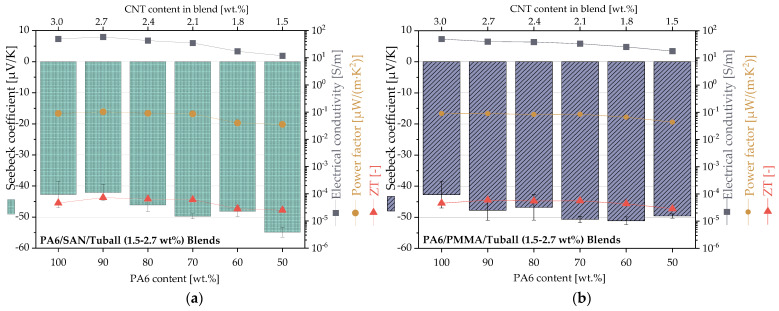
Thermoelectric parameters of (**a**) PA6/SAN/SWCNT blends and (**b**) PA6/PMMA/SWCNT blends having different ratios of the blend components and SWCNT contents (2.7–1.5 wt.%) to reach a constant SWCNT content of 3 wt.% in the PA6 component for all blend compositions (values are given in Table A2 and Table A3).

Figure 11 shows that the Seebeck coefficient becomes more negative and the volume conductivity decrease when the content of the second blend component increases and the PA6 content decreases. This tendency was found for both blend systems. The most negative Seebeck coefficient values were −54.8 μV/K for PA6/SAN/SWCNT 50/50/1.5 wt.% and −50.7 μV/K for PA6/PMMA/SWCNT 60/40/1.8 wt.%. As the increase in the absolute value of the Seebeck coefficient is comparatively lower than the decrease in electrical conductivity, this halves the value of power factor from 0.09 µW/m·K^2^ (PA6/SWCNT 100/3 wt.%) to 0.04 µW/m·K^2^ (both 50/50/1.5 wt.% blends) and reduces ZT from 4.6 × 10^−5^ (PA6/SWCNT 100/3 wt.%) to 2.5 × 10^−5^ (PA6/SAN/SWCNT 50/50/1.5 wt.%) or 2.9 × 10^−5^ (PA6/PMMA/SWCNT = 50/50/1.5 wt.%).

#### 3.6.3. Blends with 50/50 wt.% and Low SWCNT Contents

To study the influence of the SWCNT content on the thermoelectric properties, co-continuous 50/50 wt.% blends were prepared with SWCNT contents slightly above the electrical percolation threshold of 0.1 wt.% for PA6/SWCNT [65]. These blends were compared with the PA6/SWCNT composites described in [33]. Based on literature, lower CNT amounts in electrically conductive composites quite often result in higher (absolute) values of the Seebeck coefficient, whereas the electrical conductivity increases with CNT loading [33]. Thus, an optimum can be expected in the power factor and ZT values when varying the filler amount slightly above its percolation threshold.

**Figure 12:**
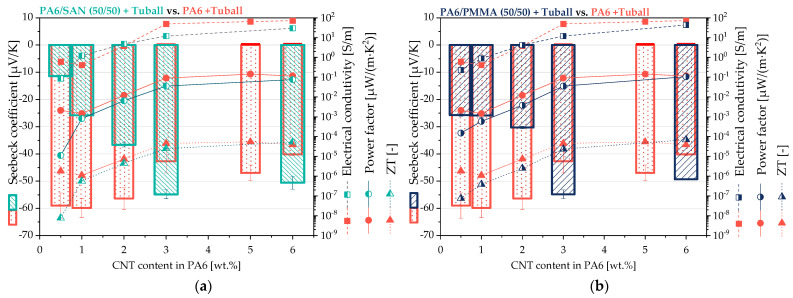
Thermoelectric parameters of (**a**) PA6/SAN/SWCNT blends and (**b**) PA6/PMMA/SWCNT blends having 50/50 wt.% ratio of the blend components and different SWCNT contents (0.25–3 wt.%) (values given in Table A2 and Table A3) in comparison to PA6/SWCNT composites (in red). The CNT contents were calculated under the assumption that the SWCNTs are completely localized in PA6.

The results in Figure 12 show that the absolute values of the Seebeck coefficients of both blend systems were significantly lower compared to PA6/SWCNT composites up to 2 wt.% SWCNT content in PA6. Starting from 3 wt.% SWCNT content in PA6 the Seebeck coefficients of both blend systems are higher than those of the corresponding PA6/SWCNT composites. The electrical volume conductivity of PA6/SWCNT composites was mainly higher than the one of the blends. Thus, in consequence the values of power factor and ZT of PA6/SWCNT composites were always slightly higher compared to the values of the blends.

## 4. Discussion

PA6 blends with PMMA or SAN were prepared which had different component ratios between 100/0 wt.% and 50/50 wt.% and SWCNT contents between 0.25 and 3 wt.%.

On the one hand, unfilled blends were produced to investigate the influence of the polymer type of the second component (PMMA, SAN) on the blend morphology. The physical properties of the polymers, such as melt viscosity and softening temperature, were very similar. An immiscible blend structure was found in which at the selected blend compositions PA6 always forms a continuous component (Figure 7 and Figure 8). In case of 50/50 wt.% blends, the rheological investigations show that the melt viscosity was between the values of the two polymers involved (Figure 2). For blends with the higher PA6 content, the viscosities were significantly closer to the values of PA6. Morphological characterizations by means of SEM have shown, as expected, an increase in the size of the dispersed particles in the matrix with increasing PMMA or SAN content. Thereby, the sizes are slightly lower in blends with PMMA than SAN which is in accordance with the slightly lower melt viscosity of PMMA.

On the other hand, SWCNTs were incorporated in PA6 and the blends. The SWCNT macro dispersion showed some large remaining agglomerates (Figure 5). Rheological measurements illustrated a significant increase in melt viscosity of the composites after SWCNT addition, which clearly exceeds the level of the unfilled polymers (Figure 3 and Figure 4). The influence of the CNT concentration can be clearly seen. The melt viscosity increases with higher SWCNT content at the same blend composition. At the same time, the blend morphology changes to a more finely distributed dispersed component (Figure 7 and Figure 8) as compared to the unfilled blends. In case of 50/50 wt.% blends, the morphology appears rather co-continuous after SWCNT addition. The quantitative analysis of the size of the dispersed component showed slightly smaller particle sizes for the blends with PMMA (Figure A2). This morphological difference is also reflected in the rheological measurements of the blend composites, where the PMMA-based composites with the finer morphology show slightly higher values of the complex melt viscosity despite the lower melt viscosity of PMMA compared to SAN.

Furthermore, the SWCNT localization in the blends is of importance. Due to the amorphous character of PMMA and SAN, these polymers soften before PA6 melts during the blend preparation by melt mixing and are therefore the first to wet the SWCNTs. However, both the morphological characterization of blends by light microscopy (Figure 6) or SEM (Figure 9) and the calculation of wetting coefficient (Table 5) have shown that the SWCNTs are always selectively located in the PA6 component. The measurements of the melt viscosity also suggest that the SWCNTs are localized exclusively in the PA6 component due to the similarity of the viscosity curves of PA6/SAN/SWCNT and PA6/PMMA/SWCNT starting at 1.5 wt.% SWCNT loading to that of the PA6 /3 wt.% composite. If the SWCNTs are all in the PA6 component, then in the 50/50 wt.% blend with 1.5 w.t% SWCNTs, there are 3 wt.% SWCNTs in the PA6 component. For both of these blend systems, the melt viscosity curves perfectly overlap with that for PA6/3 wt.% SWCNT (Figure 3 and Figure 4).

The thermal conductivity of selected samples was determined (Table 6), as it is needed for the calculation of the thermoelectric parameter figure of merit ZT. As expected the thermal conductivity of composites and blends increases with the addition of SWCNTs that have a very high thermal conductivity. However, at the same SWCNT amount, the thermal conductivity of the blends increases less compared to the PA6/SWCNT composite. In the blend there is a component with unfilled polymer with low thermal conductivity and a SWCNT-containing component with a higher thermal conductivity. The phonon transport in a blend has to take place through both good and poorly thermal conductive materials, whereby many material layers have to be bridged. This inhibits the phonon transport in the blend significantly more than in the composite, which is reflected in a lower thermal conductivity.

In set 1, 3 wt.% SWCNT were incorporated into various blends. With an electrical percolation threshold of <0.1 wt.% SWCNT in PA6 [65], it can be concluded that a dense electrical conductive network is present in the blends. Additionally, the SWCNT network density increases with increasing content of the second blend partner due to the selective SWCNT localization in PA6. With a lower PA6 content in both blend systems, the Seebeck coefficient values become more negative while the conductivity varies only slightly (Figure 10). Due to the slightly higher absolute values of the Seebeck coefficients and electrical conductivity, the power factors of PA6/SAN/3 wt.% SWCNT blends are slightly higher at 0.08–0.22 µW/m·K^2^ compared to PA6/PMMA/3 wt.% SWCNT at 0.09–0.13 µW/m·K^2^. This compares to 0.09 µW/m·K^2^ for PA6/3 wt.% SWCNT. ZT varied between 5.3 × 10^−5^ and 1.5 × 10^−4^ (PA6/SAN/3 wt.% SWCNT) or 6.4 × 10^−5^ and 8.9 × 10^−4^ (PA6/PMMA/3 wt.% SWCNT) which is slightly higher than the ZT of 4.6 × 10^−5^ for PA6/3 wt.% SWCNT.

In set 2, the CNT content in the PA6 component of the blends was kept constant at 3 wt.% so that the same network density was achieved in the electrically conductive part of the blends. With decreasing PA6 content in the blends the Seebeck coefficient values were more negative in both blend systems, however the electrical conductivity is halved (Figure 11). This leads to decreasing power factor and ZT values from 0.09 µW/m·K^2^ and 4.6 × 10^−5^ (PA6/3 wt.% SWCNT) up to 0.04 µW/m·K^2^ and 2.5 to 2.9 × 10^−5^ (PA6/PMMA or PA6/SAN with 1.5 wt.% SWCNT), respectively.

It can be summarized from the results of set 1 and 2 that the use of blends leads to changes in thermoelectric parameters such as Seebeck coefficient, power factor, and figure of merit compared to the composites having either the same SWCNT content in the whole blend or in its PA6 part. A higher SWCNT content in the PA6 achieved by increasing the content of the second component at constant 3 wt.% SWCNTs in the blends (set 1) leads to the targeted higher Seebeck coefficients at slightly varied electrical conductivity. The maximal power factors were achieved for PA6/SAN/SWCNT 70/30/3 wt.% (0.22 µW/m·K^2^) and PA6/PMMA/SWCNT 60/40/3 wt.% (0.13 µW/m·K^2^). Interestingly, these are not the compositions 50/50 at which both blends clearly form a co-continuous structure and the local concentration of SWCNTs in PA6 is the highest. At the compositions of maximal PF and ZT, both at contents of the second component lower than those which induced co-continuity (around 40–50 wt.% according to the morphological studies), the PA6 component forms the matrix structure with the second component forming dispersed particles. Thus, the local SWCNT concentration in PA6 is already increased. As soon as a co-continuous structure starts to develop obviously the continuity and integrity of the filled PA6 is disturbed in such a way, that the electrical conductivity decreases, leading to the reduction in PF and ZT. In PA6/SAN blends such disturbance of morphology and deviation from spherical dispersed particles is already achieved starting at 40 wt.% SAN (Figure 7, right column); therefore, the composition with 30 wt.% SAN leads to the best results. These results indicate the complex relationship between the blend morphology and SWCNT content on the thermoelectric parameters.

For 50/50 wt.% blends, the SWCNT concentration was further decreased to study the difference between blends and composites having the same SWCNT content in the PA6 component on their thermoelectric performance (Figure 12). For 0.5 wt.% SWCNT content in PA6, the PA6 composite showed significantly higher absolute values of Seebeck coefficient, power factor and ZT than both blends with 0.25 wt.% SWCNT. With increasing SWCNT concentration in the blends, especially the absolute value of the Seebeck coefficient was increased followed by equalization of the thermoelectric values of composites and blends starting above 3 wt.% SWCNT. However, up to 6 wt.% SWCNT in the PA6 component, always higher power factors and ZT values were observed for PA6/SWCNT composites compared to the blends, due to the lower electrical conductivity in the blends.

## 5. Conclusions

In the presented study, it was shown that the concept of segregated networks also works in immiscible PA6/SAN and PA6/PMMA blends with SWCNTs to improve thermoelectric properties. Due to the selective localization of the nanotubes in PA6 during the melt-mixing, the SWCNT network densifies with increasing amount of the second blend component. Thereby, the n-type behavior of the PA6-SWCNT composites remains after blending with SAN or PMMA. The thermoelectric properties of the blends were found to be influenced mainly by the filled matrix polymer PA6. At a constant SWCNT amount of 3 wt.% in the blends, with increasing amount of the second blend component the enrichment of SWCNTs in PA6 resulted in increasing absolute values of the Seebeck coefficient. Thereby the type of the polymer, which forms the second component in the blend (SAN or PMMA), plays only a minor role in this general effect. Thus, an increase in the power factor and ZT compared to the PA6 composite was achieved by varying the blend ratio. The maximum power factors achieved were 0.22 µW/m·K^2^ for PA6/SAN/SWCNT 70/30/3 wt.% and 0.13 µW/m·K^2^ for PA6/PMMA/SWCNT 60/40/3 wt.% compared to 0.09 µW/m·K^2^ for PA6/3 wt.% SWCNT. These maxima are not at the highest content of the second component resulting in the highest local SWCNT concentration in PA6, but occurred in the blends with the highest content of the second component still resulting in clear matrix-dispersed morphologies. The reduction of PF and ZT above these second component contents is due to a reduction in electrical conductivity which was observed as soon as co-continuous structures start to form. Blends with constant SWCNT loading in the PA6 component (lower content related to the blends with increasing part of SAN or PMMA) showed partially even higher absolute values of the Seebeck coefficients, however due to reduced electrical conductivity, PF and ZT are lower as compared to blends with higher SWCNT contents. The highest absolute S-value achieved was −54.8 μV/K for PA6/SAN/SWCNT 50/50/1.5 wt.%. This implies that there is a complex interplay between the obtained electrical conductivity and Seebeck coefficient which are differently influenced by the content of the conductive filler in the preferred blend component resulting from the content of the second component and different amounts of SWCNT addition, the SWCNT dispersion state, and the general type of blend morphology.

In context with applications of such blend systems it can be concluded that the use of polymer blends instead of single filled polymers can have advantages, as higher TE properties can be achieved at the same total filler level or the filler loading can be reduced. In addition, if an application requires the use of a blend system instead of a homopolymer, e.g., to adjust mechanical, interfacial, thermal or other properties, then part of the matrix polymer can be replaced by another polymer using the concept of segregated structures and slightly improved thermoelectric properties can be achieved at the same time.

## Figures and Tables

**Table 1:** SWCNT concentration in whole blend and in the PA6 component in dependence on blend composition.

Blend Ratio	Set 1 SWCNT Content in Whole Blend [wt.%]	Set 1 SWCNT Content in PA6 [wt.%]	Set 2 SWCNT Content in Whole Blend [wt.%]	Set 2 SWCNT Content in PA6 [wt.%]
90/10	3	3.33	2.7	3
80/20	3	3.75	2.4	3
70/30	3	4.29	2.1	3
60/40	3	5.0	1.8	3
50/50	3	6.0	1.5	3
50/50	1	2		
50/50	0.5	1		
50/50	0.25	0.5		

**Table 2:** Agglomerate area ratio for PA6/SAN/SWCNT and PA6/PMMA/SWCNT blends for sets 1 and 2.

Blend Ratio	PA6/SAN +3 wt.%	PA6/SAN +<3 wt.%	PA6/PMMA +3 wt.%	PA6/PMMA +<3 wt.%
100/0	2.5 ± 1.9		2.5 ± 1.9	
90/10	2.6 ± 1.8	0.8 ± 0.4	1.7 ± 0.5	1.0 ± 0.6
80/20	2.2 ± 1.2	0.5 ± 0.2	2.8 ± 1.0	1.4 ± 0.6
70/30	1.9 ± 0.4	1.5 ± 1.1	2.0 ± 0.5	1.0 ± 0.7
60/40	2.0 ± 0.8	1.3 ± 0.6	3.2 ± 1.1	1.3 ± 0.6
50/50	3.5 ± 1.1	1.5 ± 0.5	2.1 ± 0.5	1.7 ± 0.9

**Table 3:** Surface tensions ***γ*** of the polymers and CNTs calculated for 280 °C (processing temperature during blend preparation). Original data and references are given in Table A1.

Material ^1^	Surface Tension Total *γ* [mN/m]	Surface Tension Disperse Part *γ*^d^ [mN/m]	Surface Tension Polar Part *γ*^P^ [mN/m]
PA6	35.1	25.3	9.9
SAN 1	25.3	20.6	4.8
SAN 2	28.5	22.8	5.7
PMMA 1	23.2	18.9	4.4
PMMA 2	21.3	15.3	6.0
MWCNT 1	45.3	18.4	26.9
MWCNT 2	27.8	17.6	10.2

^1^ PA6 (Durethan B40F, Bayer; [75]), SAN 1 (unnamed; [76]), SAN 2 (SAN25 with 25 wt.% acrylonitrile in copolymer; [77]), PMMA 1 (grade EG920 from LG Chemical, South Korea; [76]), PMMA 2 (self-synthesized polymer with low molecular weight; [78]), MWCNT 1 (MWCNTs from Nanolab, USA; [73]), MWCNT 2 (MWCNTs grown by an arc-discharge method from Dynamic Enterprises, U.K.; [74]).

**Table 4:** Interfacial tensions as calculated using harmonic and geometric mean equations and values given in Table 3.

Materials	Interfacial Tension *γ*_12_, Geometric Mean Value [mN/m]	Interfacial Tension *γ*_12_, Harmonic Mean Value [mN/m]
MWCNT 1-PA6	4.72	8.96
MWCNT 2-PA6	0.69	1.37
MWCNT 1-SAN 1	9.10	15.62
MWCNT 2-SAN 1	1.14	2.22
MWCNT 1-SAN 2	8.07	14.26
MWCNT 2-SAN 2	0.99	1.94
MWCNT 1-PMMA 1	9.19	15.71
MWCNT 2-PMMA 1	1.09	2.11
MWCNT 1-PMMA 1	7.66	13.59
MWCNT 2-PMMA 1	0.64	1.26
PA6-SAN 1	1.17	2.27
PA6-SAN 2	0.63	1.24
PA6-PMMA 1	1.49	2.90
PA6-PMMA 2	1.71	3.37

**Table 5:** Wetting coefficients as calculated using harmonic and geometric mean equations.

Composition	Wetting Coefficient *ω*a, Geometric Mean Value [−]	Wetting Coefficient *ω*a, Harmonic Mean Value [−]
PA6/SAN 1/MWCNT 1	3.76	2.93
PA6/SAN 1/MWCNT 2	0.39	0.37
PA6/SAN 2/MWCNT 1	5.3	4.26
PA6/SAN 2/MWCNT 2	0.47	0.46
PA6/PMMA 1/MWCNT 1	3.01	2.32
PA6/PMMA 1/MWCNT 2	0.27	0.25
PA6/PMMA 2/MWCNT 1	1.72	1.38
PA6/PMMA 2/MWCNT 2	−0.03	−0.03

**Table 6:** Results of thermal conductivity measurements of different samples.

Sample	Density [g/cm^3^]	Temperature Conductivity [mm^2^/s]	Specific Heat Capacity [J/g·K]	Thermal Conductivity [W/m·K]
PA6	1.12	0.17 ± 0.03	1.62 ± 0.08	0.31 ± 0.04
SAN	1.05	0.13 ± 0.00	1.50 ± 0.01	0.20 ± 0.00
PMMA	1.11	0.14 ± 0.00	1.58 ± 0.00	0.25 ± 0.00
PA6/SAN 50/50	1.05	0.16 ± 0.01	1.65 ± 0.03	0.27 ± 0.02
PA6/PMMA 50/50	1.12	0.16 ± 0.00	1.50 ± 0.00	0.27 ± 0.00
PA6 +3 wt.% SWCNT	1.16	0.36 ± 0.01	1.48 ± 0.01	0.62 ± 0.03
PA6/SAN 50/50 +3 wt.% SWCNT	1.11	0.27 ± 0.01	1.47 ± 0.05	0.45 ± 0.01
PA6/PMMA 50/50 +3 wt.% SWCNT	1.17	0.28 ± 0.01	1.45 ± 0.02	0.48 ± 0.02

## Data Availability

The data presented in this study are available on request from the corresponding author.

## Appendix A

**Table A1:** Surface tension values of the polymers and CNTs as taken from the literature.

Material	Surface Tension Total *γ* [mN/m]	Surface Tension Disperse Part *γ*^d^ [mN/m]	Surface Tension Polar Part *γ*^P^ [mN/m]	Temperature Coefficient d*γ*/dT [mN/m·°C]	Polarity [%]
PA6	36.1 @ 265 °C [80]			−0.065 [81]	28 [81]
PA6	37.7 @ 240 °C [75]	27.2 [75]	10.6 [75]		28 *
SAN 1 [76]	45.3 @ 23 °C 33.1 @ 180 °C	36.8 @ 23 °C	8.5 @ 23 °C	−0.078 *	19 *
SAN 2	29.0 @ 270 °C, 30.0 @ 250 °C [77]				24 [68]
PMMA 1 [76]	40.5 @ 23 °C 29.9 @ 180 °C	32.4 @ 23 °C	8.1 @ 23 °C	−0.067 *	20 *
PMMA 2	28.9 @ 180 °C [78]			−0.076 [78]	28 [82]
MWCNT 1 [73]	45.3	18.4	26.9		59 *
MWCNT 2 [74]	27.8	17.6	10.2		37 *

* Calculated from given absolute values.

**Table A2:** TE parameters of PA6/SAN/SWCNT blends.

PA6/SAN/SWCNT	Volume Conductivity [S/m]	Seebeck Coefficient [µV/K]	Power Factor [µW/m·K^2^]	ZT [−]
100/0/6	71.6	−40.2 ± 0.4	0.1157	3.9 × 10^−5 1^
100/0/3	49.3	−42.7 ± 4.3	0.0899	4.6 × 10^−5 2^
90/10/3	67.1	−53.0 ± 5.3	0.1884	1.3 × 10^−4 3^
80/20/3	44.6	−48.5 ± 0.7	0.1049	7.3 × 10^−5 3^
70/30/3	88.0	−50.0 ± 2.1	0.2165	1.5 × 10^−4 3^
60/40/3	42.1	−51.7 ± 1.6	0.1127	7.9 × 10^−5 3^
50/50/3	29.5	−50.6 ± 2.3	0.0757	5.3 × 10^−5 3^
90/10/2.7	58.5	−42.1 ± 2.6	0.1035	7.2 × 10^−5 3^
80/20/2.4	42.9	−49.1 ± 2.1	0.0912	6.4 × 10^−5 3^
70/30/2.1	35.1	−49.7 ± 0.8	0.0867	6.1 × 10^−5 3^
60/40/1.8	17.4	−48.1 ± 1.7	0.0402	2.8 × 10^−5 3^
50/50/1.5	11.8	−54.8 ± 1.6	0.0356	2.5 × 10^−5 3^
50/50/1	4.8	−36.7 ± 0.8	0.0065	4.6 × 10^−6 3^
50/50/0.5	1.2	−25.8 ± 1.2	0.0008	5.7 × 10^−7 3^
50/50/0.25	0.1	−11.4 ± 0.2	1.1 × 10^−5^	7.8 × 10^−9 3^

^1^ thermal conductivity 0.93 W/m·K (extrapolated from value of PA6/3 wt.% SWCNT); ^2^ thermal conductivity 0.62 W/m·K; ^3^ thermal conductivity 0.45 W/m·K (compare Table 6).

**Table A3:** TE parameters of PA6/PMMA/SWCNT blends.

PA6/PMMA/SWCNT	Volume Conductivity [S/m]	Seebeck Coefficient [µV/K]	Power Factor [µW/m·K^2^]	ZT [−]
100/0/3	49.3	−42.7 ± 4.3	0.0899	4.6 × 10^−5 1^
90/10/3	79.2	−41.2 ± 2.0	0.1347	8.9 × 10^−4 2^
80/20/3	42.2	−48.0 ± 2.7	0.0973	6.4 × 10^−5 2^
70/30/3	39.6	−48.5 ± 0.5	0.0931	6.1 × 10^−4 2^
60/40/3	65.2	−44.7 ± 2.2	0.1304	8.6 × 10^−5 2^
50/50/3	43.4	−49.3 ± 0.2	0.1056	7.0 × 10^−5 2^
90/10/2.7	39.4	−47.7 ± 3.2	0.0898	5.9 × 10^−5 2^
80/20/2.4	38.2	−46.8 ± 4.1	0.0836	5.5 × 10^−5 2^
70/30/2.1	33.2	−50.7 ± 1.0	0.0853	5.6 × 10^−5 2^
60/40/1.8	25.3	−51.1 ± 1.2	0.0662	4.4 × 10^−5 2^
50/50/1.5	17.7	−49.6 ± 0.7	0.0436	2.9 × 10^−5 2^
50/50/1	4.2	−30.3 ± 0.4	0.0038	2.5 × 10^−6 2^
50/50/0.5	0.9	−25.9 ± 0.8	0.0006	3.9 × 10^−7 2^
50/50/0.25	0.2	−25.7 ± 0.4	0.0002	7.7 × 10^−8 2^

^1^ thermal conductivity 0.62 W/m·K; ^2^ thermal conductivity 0.48 W/m·K (compare Table 6).
